# Obstetrics Outcomes in Women Undergoing Second-Stage Cesarean Section: A Cross-Sectional Study

**DOI:** 10.7759/cureus.39911

**Published:** 2023-06-03

**Authors:** Charmy A Vashi, Nikita Vijay, Anuja Bhalerao, Anushree Shetty

**Affiliations:** 1 Department of Obstetrics and Gynaecology, NKP Salve Institute of Medical Sciences and Research Centre, Nagpur, IND

**Keywords:** vaginal delivery, post partum haemorrhage, second stage of labor, obstetric outcome, caesarean section

## Abstract

Background

Cesarean section (CS) involves the delivery of the fetus through incisions in the abdomen or uterine walls which is an alternative to vaginal delivery. In the majority of women, second-stage CS is performed without even attempting assisted vaginal delivery. This leads to difficulty for obstetricians for whether to perform an immediate CS or attempt a difficult vaginal birth as the effective method of delivery as CS are linked with higher morbidities which further increase when a CS is performed in the second stage. Hence, the present study was performed to find out obstetrics results in women undergoing second-stage CS.

Method

A cross-sectional study was conducted in the Department of Obstetrics and Gynecology of a tertiary care center attached to a medical college to study obstetric outcomes in women undergoing second-stage CS from January 2021 to December 2022 on 54 postnatal women who underwent second-stage CS.

Results

The mean age was 26.7 ± 3.9 years ranging between 19 and 35 years involving a majority of primiparity women. Maximum patients were having gestational age between 39 and 40 weeks and had spontaneous labor. The main indication of second-stage CS was non-reassuring fetal status and the method of delivery mainly involved for the deeply impacted head was the modified patwardhan technique in which if the head is embedded deeply in the pelvis in the OP position the anterior shoulder is delivered first followed by the same side leg then opposite side leg followed by the arm is gently delivered. Baby's trunk, legs, and buttocks are moved out by pulling them carefully and gently. And lastly, the head of the infant is finally moved out. The intra-operative complications mainly found were an extension of uterine angle and the post-operative complication was post-partum hemorrhage (PPH). The most common neonatal complication was the requirement for neonatal intensive care unit (NICU) admission. In conclusion, the present study reported a hospital range between seven and 14 days in comparison to other studies that reported hospital stays between three and 15 days.

Conclusion

In conclusion, higher maternal and fetal morbidities were associated with CS performed at full dilation of the cervix. The most common maternal complication seen was an injury to uterine vessels along with PPH however neonatal complications included the requirement of NICU monitoring. As there are no appropriate guidelines for the same, formulation of guidelines for performing CS at full dilation is required.

## Introduction

Cesarean section (CS) is the delivery of the fetus through incisions in the abdominal and uterine walls and is considered an alternative to vaginal delivery [1-3]. Recent data demonstrate that the prevalence of CS is increasing rapidly with second-stage CS from 0.9% to 2.2% [4,5]. The second stage of labor which usually used to last two hours begins with full cervical dilation that is 10 cm and completes with the delivery of the fetus [6,7]. The duration is recently increased due to regional anesthesia [3,4]. According to the Royal College of Obstetricians and Gynecologists (RCOG), 6% of primary CS occur at full cervical dilatation [8,9].

In the majority of women, second-stage CS is performed without even attempting assisted vaginal delivery [4,10]. This leads to difficulty for obstetricians for whether to perform an immediate CS or attempt a difficult vaginal birth as the effective method of delivery [11]. Additionally, in comparison to vaginal deliveries, CS is linked with higher morbidities which further increase when a section is performed in the second stage as the head of the fetus is deeply impacted into the pelvis, and extraction is technically difficult to perform. Moreover, complications are limited not only to the mother but also to the fetus [8,12].

Maternal complications which can be encountered are tearing of the lower part of the uterus and surrounding structures, as well as uterine angle extension, broad ligament hematoma, postpartum hemorrhage, bladder injury, hematuria, puerperal pyrexia, infections, extended hospitalization, and catheterization. Birth-related problems in neonates like asphyxia, injuries, fetal academia, hypoxic-ischemic encephalopathy, prolonged NICU stay, and even stillbirths are common [9,13]. Therefore, in an attempt of reducing the deliveries through CS and its associated complications, the American College of Obstetricians and Gynecologists (ACOG) has extended the duration of the second stage of labor [9,13,14].

For performing CS following complete dilation of the cervix there are no accurate guidelines. Either emergency or an elective CS, both are more likely to experience complications than a typical vaginal delivery. Owing to the complexity of CS in the second stage of labor, it is technically challenging to accomplish due to the profoundly impacted head of the fetus in the pelvis, the lack of liquor, and the occurrence of the oedematous thin segment at a lower level [5,9,12].

In spite of the rise in CS during the last 20 years, the escalation of emergencies has received little attention for CS in the second stage. For many women, CS is either unplanned or not even considered, and it’s disappointing and traumatic for the women when CS is done after a very long and difficult second stage of labor (that is at complete cervical dilatation) [5,9,12]. Training gaps, inadequate monitoring of junior staff when making decisions, and an absence of national guidelines for conducting sections at full dilation are among the significant factors contributing to the alarming increase in CS deliveries [5,9,15]. Therefore making a decision in terms of CS in the second stage of labor is a major issue in modern obstetrics practice. To establish ideal management, guidelines, practice for structured training programs, and specific drilling for managing safe delivery are required. Hence, the present study was performed to find out obstetrics results in women undergoing second-stage CS.

## Materials and methods

A cross-sectional study was conducted in the Department of Obstetrics and Gynecology of a tertiary care center attached to a medical college to study obstetric outcomes in women undergoing second-stage CS. After obtaining permission from the institutional ethics committee (IEC) with reference number 103/2021 and written informed consent from the women, the study was carried out from January 2021 to December 2022 on 54 postnatal women who underwent second-stage CS. The women were analyzed in terms of whether the labor was spontaneous or induced, duration of labor, per speculum, and per vaginal findings at the time when the decision for CS was taken which involved cervicovaginal infection, color and amount of liquor, station of the head, molding, caput, and rotation of the head. Additionally, the attempt for instrumental delivery, indication for CS, anesthesia types, abdominal and uterine incision, the technique of fetal head removal, and incision to delivery time were analyzed.

Furthermore, intraoperative complications like an extension of uterine angles leading to broad ligament hematoma, bladder injury, tearing of lower uterine segment and an adjacent structure, bowel injury, and excessive blood loss along with the status of the fetus consisting of APGAR score at 1 and 5 min, baby weight, Neonatal Intensive Care Unit (NICU) admission and indication for admission, birth asphyxia, injuries to baby, sepsis, duration of hospitalization, and neonatal death were analyzed. Postoperative complications involving postpartum hemorrhage, postpartum pyrexia, postpartum infection, wound infection, and duration of catheterization and hospitalization were also analyzed.

Data was stored in MS Excel for analysis. Statistical Package for Social Sciences (SPSS ver. 24.0, IBM Corporation, USA) for MS Windows was used to evaluate the data, and data on continuous variables were provided as mean and standard deviation (SD), whereas the data on categorical variables were displayed as n (% of cases).

## Results

**Figure 1:**
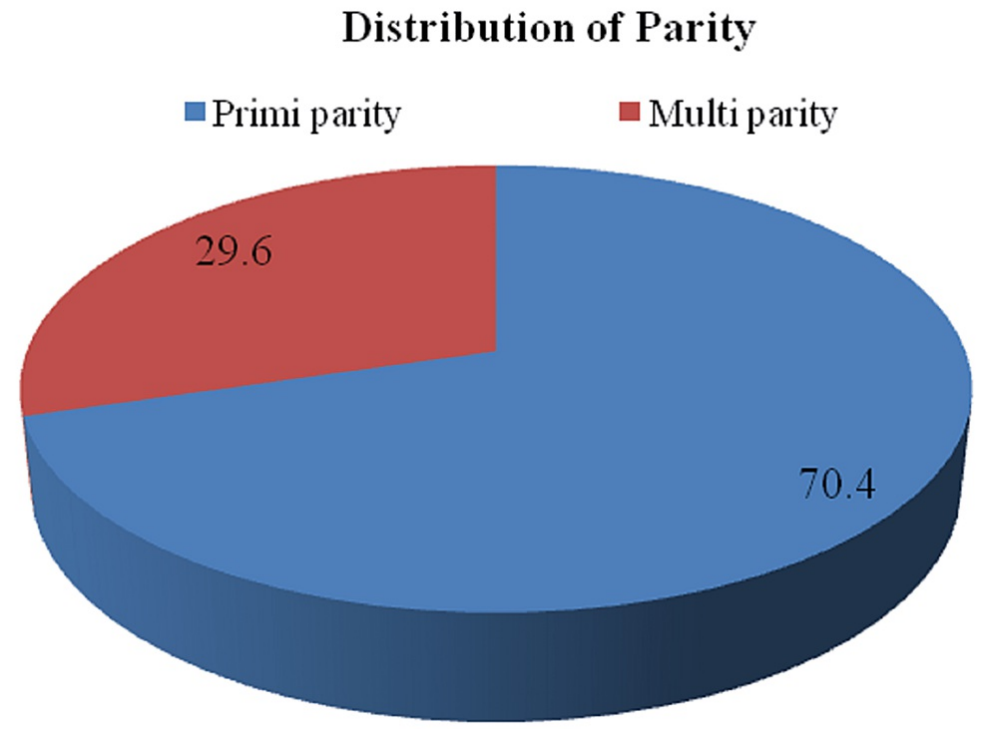
Distribution of women according to parity

The present cross-sectional observational study was performed to study the obstetrics outcome in women undergoing second-stage CS. A total of 54 women (cases) were included in the study based on the criteria. Of 54 cases studied, 12 (22.2%) had an age between 19 and 23 years, 25 cases (46.3%) had an age between 24 and 28 years, 16 cases (29.6%) had an age between 29 and 33 years, and one case (1.9%) had age more than 33 years. The mean age of patients studied was 26.7 ± 3.9 years. The distribution of socio-economic status (SES) among the cases studied demonstrated that four cases (7.4%) had lower SES, 25 cases (46.3%) had lower middle SES, 15 cases (27.8%) had lower upper SES, six cases (11.1%) had upper lower SES, and four cases (7.4%) had upper middle SES in the study group. Additionally, the distribution of women according to parity is illustrated in Figure 1.

**Figure 2:**
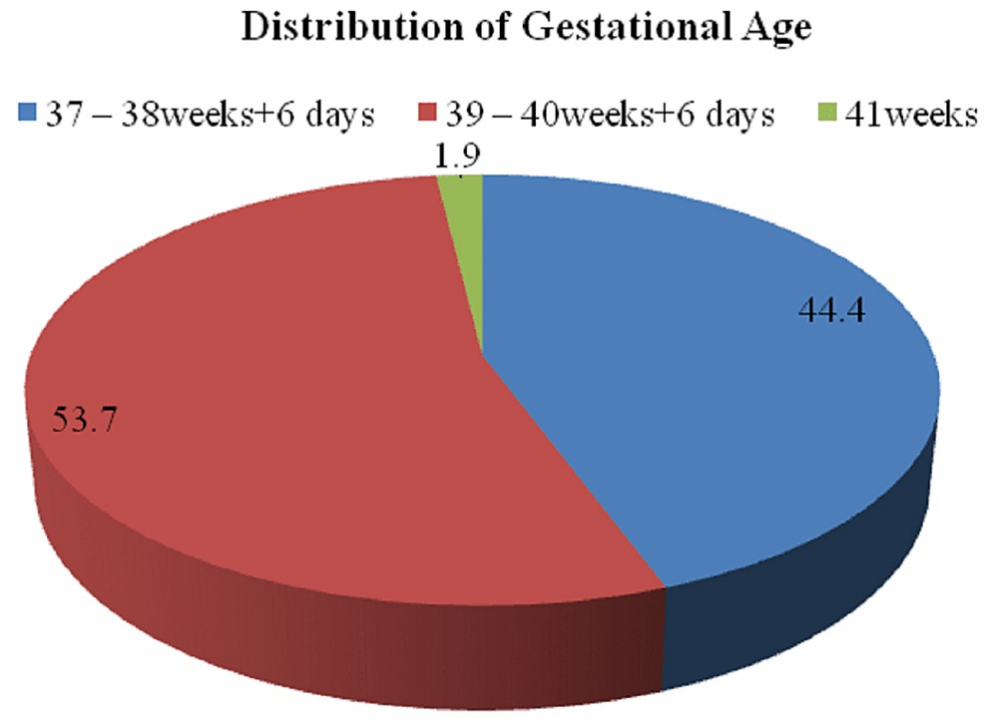
Distribution of gestational age among the cases studied

The distribution of patients according to the gestational age is depicted in Figure 2.

**Figure 3:**
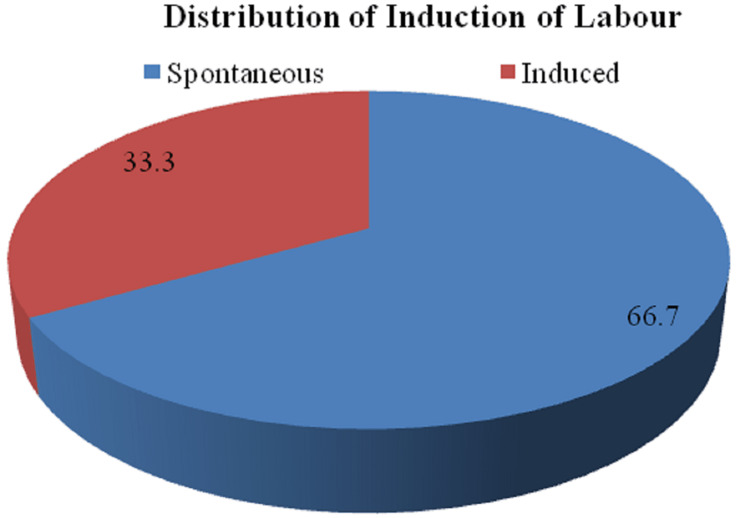
Distribution of patients according to induction of labor

The distribution of patients according to induction of labor is illustrated in Figure 3.

**Figure 4:**
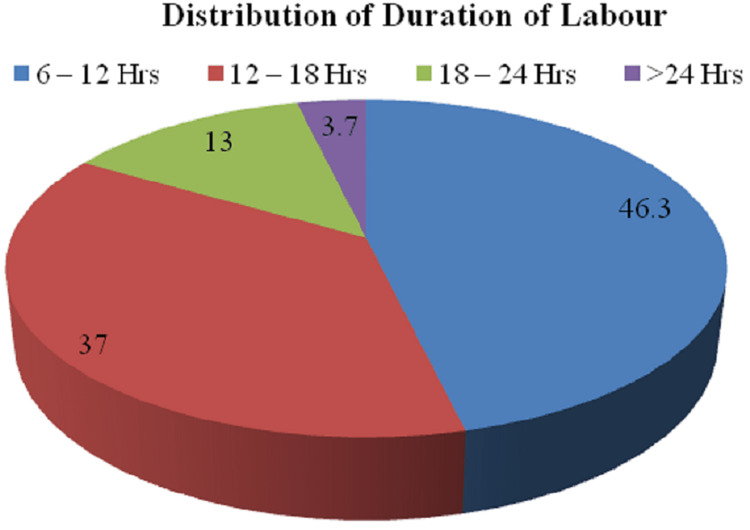
Distribution of patients according to the duration of labor

The distribution of patients according to the duration of labor is depicted in Figure 4.

The distribution of patients according to indications of second-stage CS is demonstrated in Table 1.

**Table 1:** Distribution of patients according to indications of second-stage CS CS: cesarean section

Indications	No. of cases	% of cases
Non-reassuring fetal status	19	35.2
Non-progress of labor	11	20.4
Cephalopelvic disproportion (CPD)	10	18.5
Deep transverse arrest (DTA)	7	13.0
Failed operative vaginal delivery	4	7.4
Occipito-posterior	3	5.6
Total	54	100.0

The distribution of patients according to the method of delivery of deeply impacted head consisting of modified Patwardhan, vertex, Patwardhan, and push method [16] is described in Table 2.

**Table 2:** Distribution according to the method of delivery of the deeply impacted head

Method of delivery [16]	No. of cases	% of cases
Modified Patwardhan	19	35.2
Vertex	15	27.8
Patwardhan	11	20.4
Push	9	16.7
Total	54	100.0

The distribution of patients according to the incision to delivery time among the cases studied is demonstrated in Table 3.

**Table 3:** Distribution of patients according to the incision to delivery time among the cases studied

Incision to delivery time	No. of cases	% of cases
3-3min+59sec	8	14.8
4-4min+59sec	12	22.2
5-5min+59sec	15	27.8
6-6min+59sec	17	31.5
7-7min+59sec	2	3.7
Total	54	100.0

The distribution of patients according to the incidence of intra-operative complications is described in Table 4.

**Table 4:** Distribution of patients according to the incidence of intra-operative complications PPH: post-partum hemorrhage

	Present		Absent		Total	
Intra-operative complications	n	%	n	%	n	%
Atonic PPH	7	13.0	47	87.0	54	100.0
Extension of uterine angle	11	20.4	43	79.6	54	100.0
Injury to uterine vessels	3	5.6	51	94.4	54	100.0
Blood transfusion	9	16.7	45	83.3	54	100.0
Bladder injury	1	1.9	53	98.1	54	100.0
Blood-stained urine	6	11.1	48	88.9	54	100.0

The distribution of patients according to the incidence of postoperative complications is demonstrated in Table 5.

**Table 5:** Distribution of patients according to the incidence of post-operative complications

	Present		Absent		Total	
Post-operative complications	n	%	n	%	n	%
Febrile illness	3	5.6	51	94.4	54	100.0
Wound infection	3	5.6	51	94.4	54	100.0
Postpartum hemorrhage	4	7.4	50	92.6	54	100.0

The distribution of patients showing the incidence of neonatal complications is described in Table 6.

**Table 6:** Distribution of patients showing the incidence of neonatal complications NICU: Neonatal Intensive Care Unit

	Present		Absent		Total	
Neonatal complications	n	%	n	%	n	%
1 Min APGAR <5	6	11.1	48	88.9	54	100.0
5 Min APGAR <5	7	13.0	47	87.0	54	100.0
Respiratory distress	9	16.7	45	83.3	54	100.0
Birth injuries	2	3.7	52	96.3	54	100.0
NICU admission	16	29.6	38	70.4	54	100.0
Septicemia	6	11.1	48	88.9	54	100.0
Fresh Stillbirth	1	1.9	53	98.1	54	100.0

**Figure 5:**
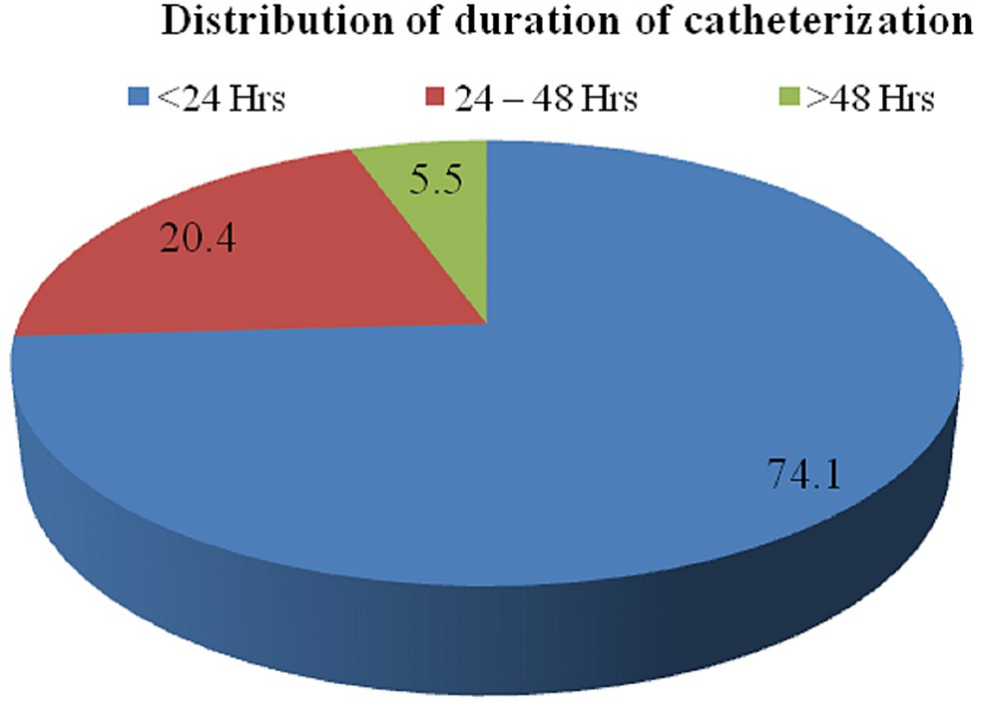
Distribution of patients according to the duration of catheterization

The distribution of patients according to the duration of catheterization is demonstrated in Figure 5.

**Figure 6:**
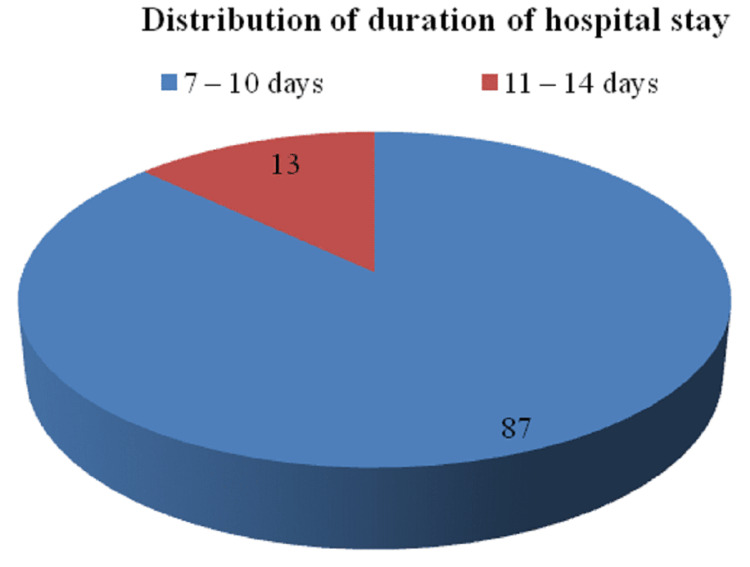
Distribution of patients according to the duration of hospital stay

The distribution of patients according to the duration of hospital stay is depicted in Figure 6.

## Discussion

The present study was conducted in the Department of Obstetrics and Gynaecology in a tertiary care hospital attached to a medical college, the total number of women included in this study was 54 and the mean age was 26.7 ± 3.9 years ranging between 19 and 35 years. Similarly, in a study given by Moodley et al., the mean age of cases studied was 23.79 ± 5.7 [6]. Additionally, Govender et al. reported mean age of 25.2 ranging between 13 and 48 years [15]. The distribution of patients according to their parity described that 38 (70.4%) patients were of primiparity and 16 (29.6%) patients were of multiparity. Similarly, Babre et al. concluded a maximum number of patients were of primigravida 45 (74%), and only 16 (26%) were multigravida [16].

The distribution of patients according to their gestational age reported that of a total of 54 patients, a maximum of patients 29 (53.7%) had a gestational age between 39 and 40 weeks + six days. Incongruous with the above study, a study given by Anusha, in 40 deliveries, the gestational age ranged from 37 to 39 weeks, with only three deliveries exceeding 40 weeks [9]. The distribution depending on the induction of labor demonstrated that out of a total of 54 patients, 36 (66.7%) patients had spontaneous labor, and 18 (33.3%), patients had induced labor in the study group. Additionally, out of 54 patients, 25 (46.3%) patients had a duration between 6 and 12 hours, 20 (37.0%) had a duration between 12 and 18 hours, seven (13.0%) patients had a duration between 18 and 24 hours, and two (3.7%) had duration above 24 hours. Furthermore, a study given by Unterscheider et al. that compared induced labor characteristics from the year 2006-2008 demonstrated 10 (34.5%) patients in 2006, 18 (41.9%) in 2007, and 21 (32.8%) in 2008 constituting a total of 49 (36%) patients [13].

The distribution of patients according to indications of second-stage CS reported that out of 54 cases studied, 19 cases (35.2%) had non-reassuring fetal status, 11 cases (20.4%) had non-progress of labor, 10 cases (18.5%) had CPD, seven cases (13.0%) had DTA, four cases (7.4%) had failed operative vaginal delivery, and three cases (5.6%) had occipito-posterior in the study group. According to Babre et al., the most frequent signs were non-descending head, deflexed head, DTA, failed vacuum, and occipito-posterior [16]. In research presented by Belay et al., CPD (48.5%) was the most prevalent indication [17]. According to a study by Kumaresan et al., CPD which affected 87 patients (34.8%) was the most common reason for a second-stage CS [18]. Furthermore, according to Goswami et al., DTA seven (14%) and deflexed head eight (16%) were the two most frequent indications for LSCS in the second stage of labor, accounting for 19 (38%) of the cases [19].

The distribution of patients according to the method of delivery of deeply impacted head described that out of 54 patients, 19 (35.2%) had the Modified Patwardhan method, 15 cases (27.8%) had the vertex method used, 11 cases (20.4%) had Patwardhan method, and nine cases (16.7%) had push method used for deeply impacted head in the study group. Whereas, distribution of patients in accordance with an incision to delivery time described that eight cases (14.8%) had incision to delivery time between 3 and 3min+59 sec, 12 cases (22.2%) had incision to delivery time between 4 and 4min+59 sec, 15 cases (27.8%) had incision to delivery time between 5 and 5min+59 sec, 17 cases (31.5%) had incision to delivery time between 6 and 6min+59 sec, and two cases (3.7%) had incision to delivery time between 7 and 7min+59 sec in the study group.

Similarly, Goswami et al. reported that the most used method of delivery of deeply impacted head was Patwardhan (50%), followed by vertex (36%), push method (6%), foot and breech method with 4% each [19]. Additionally, according to Babre et al., the most used method of delivery was vertex 41 (67.2%), followed by Patwardhan 14 (23%), and push method six (9.8%) [16]. Furthermore, by Kumaresan et al. the most common method of delivery is the Patwardhan technique 112 (44.8%), push method 68 (27.2%), conventional method 43 (17.2%), and reverse breech extraction (pull method) 27 (10.8%) [18].

The distribution of patients according to the incidence of intra-operative complications showed that out of 54 patients, seven (13.0%) had atonic postpartum hemorrhage (PPH), 11 cases (20.4%) had an extension of uterine angle, three cases (5.6%) had an injury to uterine vessels, nine cases (16.7%) required blood transfusion, one case (1.9%) had bladder injury, and six cases (11.1%) had blood stained urine in the study group. All complications were non-exclusive, meaning a few cases had multiple intra-operative complications. The incidence of post-operative complications demonstrated that out of 54 cases studied, three cases (5.6%) had febrile illness, three cases (5.6%) had wound infection, and four cases (7.4%) had PPH in the study group. All complications were non-exclusive, meaning a few cases had multiple post-operative complications.

In a study conducted by Khaniya et al., 20/36 patients experienced intraoperative complications. Blood-stained urine was the most common, occurring in 14 patients (33.88%), followed by uterine incision extension in five patients (13.88%), and B lynch compression suture was encountered in only one woman (2.77%) with atonic PPH [5]. By Anusha SR et al. the PPH in 37 (74%) patients was the main complication, followed by a blood transfusion that involved 29 (58%) patients, and the incidence of uterine tear in eight (16%) patients [9]. Additionally, in a study given by Goswami et al. extension of uterine angles that involved eight (16%) patients was the main complication, followed by atonic PPH involving four (8%) patients, bladder injuries involving three (6%) patients, and obstetric hysterectomy including two (4%) patients [19]. Furthermore, in a study given by Moodley et al., the postoperative complication involved early PPH that involved three patients, and four patients were there for post-operative fever [6].

Govender et al. in their study mentioned the maternal complications in the second stage that involved PPH consisting of five (4.6%) patients, wound sepsis four (3.5%), blood transfusion six (5.2%), blood loss 17 (14.7%), post-operative fever 34 (29.3%), hysterectomy two (1.7%), stepwise devascularization one (0.9%), compression suture three (2.5%), extension tear on uterus 26 (22.4%), lower segment tear 11 (9.48%), bladder injury four (3.5%), fetus's head pushed up 86 (74.1%), relaparotomy with two (1.72%), puerperal infection two (1.7%) [15]. A study given by Jayaram et al. demonstrated four maternal complications of PPH accounting for five (19.23%) patients, PPH (surgical management) involving two (7.69%), lower uterine section tear and angle extension consisting of four (15.38%), febrile morbidity four (15.38%), blood-stained urine five (19.23%), and wound sepsis one (3.84%) [3]. In conclusion, the most common maternal complication seen was an injury to uterine vessels along with PPH in the present study, whereas, in other studies, PPH, blood-stained urine, fetus's head pushed up, and extension of uterine angle were the most common complications.

Additionally, the distribution of patients according to the incidence of neonatal complications demonstrated that out of 54 babies delivered, six babies (11.1%) had 1 min APGAR score below 5, eight babies (13.0%) had 5 min APGAR score below 5, nine babies (16.7%) had respiratory distress, three babies (3.7%) had birth injuries, 17 babies (29.6%) required NICU admission, one baby (1.9%) had a stillbirth, and none of the babies had neonatal death in the study group. All complications were non-exclusive, meaning a few babies had multiple complications. A study given by Gurung et al. demonstrated seven fetal and newborn complications with Meconium stained liquor consisting of 49 (34.2%) neonates, admission to nursery consisting of 22 (15.3%), NICU admission involving five (3.4%), neonatal jaundice in 14 (9.7%), Cephalhematoma in two (1.3%), Apgar score < 7 at 5 min including 13 (9%), and fresh stillbirth involving one (0.6%) [11]. Khaniya et al. showed perinatal outcomes in their study and reported that baby weight between 2.5 and 2.9 involved two (5.5%) neonates, 3-3.5 involved four (11.11%), between 3.6 and 4.0 consisting of 30 (83.33%) neonates, meconium stain liquor with 10 (27.77%), Apgar score<5 at 5 min including five (13.88%), NICU admission with two (5.55%) neonates, and fresh stillbirth consisting of one (2.77%) neonates [5]. In conclusion, the most common neonatal complication seen in the present study was the requirement of NICU admission accounting for 17 babies (29.6%). Whereas, the other studies reported meconium stain liquor to be the common complication.

Furthermore, the distribution of patients according to the duration of catheterization described that of a total of 54 cases studied, 40 cases (74.1%) had a duration <24 hrs, 11 cases (20.4%) had a duration between 24 and 48 hrs and three cases (5.5%) had more than 48 hrs in the study group. Additionally, the duration of hospital stay among the cases studied demonstrated that of 54 cases studied, 47 cases (87.0%) had a stay between seven and 10 days, and seven cases (13.0%) had a duration between 11 and 14 days.

In a study given by, Markandu et al. mean hospital stay was 2.28 days [10]. Additionally, in a study given by Gurung et al., it was 5.59 days [11]. Furthermore, a study given by Moodley et al. reported four days ranging between three and 15 days, and similarly, the length of neonatal hospital stay was of four days ranging between two and 10 days [6]. In conclusion, the present study reported a hospital range between seven and 14 days in comparison to other studies that reported hospital stays between three and 15 days. The limitations of the study mainly consisted of shorter duration of the study period and small sample size which may be overcome in future studies to highlight more prominent obstetric outcomes of second-stage CS. Additionally, the time interval between the second stage of labor to the decision for cesarean and its relevance with intraoperative and postoperative complications can be considered for the future scope of the study.

## Conclusions

The present study aimed to study the obstetrics outcome in women undergoing second-stage cesarean section and concluded that there are higher maternal and fetal morbidities when a section is performed at full dilation of the cervix. The most common maternal complication seen was an injury to uterine vessels along with PPH however neonatal complications included the requirement of NICU monitoring. Currently, there is a tremendous rise in the rates of second-stage CS and there are no appropriate guidelines for conducting the same which potentiates the need for conducting this study. Lastly, formulation of guidelines for performing CS at full dilation is required, especially in this field where a lot of medico-legal issues are involved ultimately impacting the lives of the mother and the fetus.
